# mRNA Vaccines Against SARS‐CoV‐2 Variants Delivered by Lipid Nanoparticles Based on Novel Ionizable Lipids

**DOI:** 10.1002/adfm.202204692

**Published:** 2022-07-19

**Authors:** Kepan Chen, Na Fan, Hai Huang, Xin Jiang, Shugang Qin, Wen Xiao, Qian Zheng, Yupei Zhang, Xing Duan, Zeyi Qin, Yongmei Liu, Jun Zeng, Yuquan Wei, Xiangrong Song

**Affiliations:** ^1^ Department of Critical Care Medicine Frontiers Science Center for Disease‐related Molecular Network State Key Laboratory of Biotherapy West China Hospital Sichuan University Chengdu 610041 China; ^2^ Department of Biology Brandeis University Boston MA 02453 USA

**Keywords:** ionizable lipids, lipid nanoparticles, mRNA delivery, mRNA vaccines, SARS‐CoV‐2 variants

## Abstract

SARS‐CoV‐2 variants are now still challenging all the approved vaccines, including mRNA vaccines. There is an urgent need to develop new generation mRNA vaccines with more powerful efficacy and better safety against SARS‐CoV‐2 variants. In this study, a new set of ionizable lipids named 4N4T are constructed and applied to form novel lipid nanoparticles called 4N4T‐LNPs. Leading 4N4T‐LNPs exhibit much higher mRNA translation efficiency than the approved SM‐102‐LNPs. To test the effectiveness of the novel delivery system, the DS mRNA encoding the full‐length S protein of the SARS‐CoV‐2 variant is synthesized and loaded in 4N4T‐LNPs. The obtained 4N4T‐DS mRNA vaccines successfully trigger robust and durable humoral immune responses against SARS‐CoV‐2 and its variants including Delta and Omicron. Importantly, the novel vaccines have higher RBD‐specific IgG titers and neutralizing antibody titers than SM‐102‐based DS mRNA vaccine. Besides, for the first time, the types of mRNA vaccine‐induced neutralizing antibodies are found to be influenced by the chemical structure of ionizable lipids. 4N4T‐DS mRNA vaccines also induce strong Th1‐skewed T cell responses and have good safety. This work provides a novel vehicle for mRNA delivery that is more effective than the approved LNPs and shows its application in vaccines against SARS‐CoV‐2 variants.

## Graphical abstract

In this study, mRNA vaccines against SARS‐CoV‐2 variants delivered by lipid nanoparticles based on 4N4T lipids are constructed, and successfully trigger robust and durable humoral immune responses against SARS‐CoV‐2 and its variants including Delta and Omicron. In addition, head‐to‐head comparison studies find that the novel 4N4T lipids have a higher mRNA delivery efficiency than SM‐102.

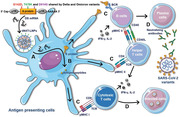

## 1 Introduction

The sudden appearance and rapid pandemic of Coronavirus disease 2019 (COVID‐19) caused by severe acute respiratory syndrome coronavirus 2 (SARS‐CoV‐2) seriously endangered human health and social development. To date, a vaccine is still the most powerful strategy to prevent COVID‐19.^[^ ^1^, ^2^ ^]^ Since the outbreak of COVID‐19, mRNA vaccines are leading the race of vaccines against SARS‐CoV‐2 on account of their advantages of rapid development, good safety, and broad immune responses.^[^ ^3^, ^4^, ^5^ ^]^ Recently, the Food and Drug Administration (FDA) approved the biologics licensing application (BLA) submitted by Moderna for SPIKEVAX (mRNA‐1273), which is the second approved mRNA vaccine after COMIRNATY (BNT162b2 of Pfizer/BioNTech). The mRNA vaccines definitely played an important role in the prevention of COVID‐19; however, both efficacy and safety of the approved mRNA vaccines have been questioned, especially facing the emerging variants of SARS‐CoV‐2.^[^ ^6^, ^7^, ^8^ ^]^ As the epidemic of COVID‐19 continues to expand, an increasing number of variants of SARS‐CoV‐2 with various mutations are emerging and have replaced the wild‐type, especially the Variants of Concern (VOC), such as Delta (B.1.617.2) and Omicron (B.1.1.529). The Omicron variant discovered in South Africa on November 9, 2021, has now replaced the Delta variant as the globally dominant strain. The mutations in the spike protein (S) give the variants the ability of immune evasion, for example, L452R, T478K, and D614G.^[^ ^9^, ^10^, ^11^ ^]^ Thus, the variants are now challenging the first‐generation vaccines developed against wild‐type SARS‐CoV‐2, such as BNT162b2 encoding the S protein of wild‐type SARS‐CoV‐2.^[^ ^12^, ^13^, ^14^ ^]^ Multiple SARS‐CoV‐2 variants have escaped neutralization by vaccine‐induced humoral immunity.^[^ ^6^, ^15^, ^16^, ^17^, ^18^ ^]^ Besides, there are also concerns about the safety of the approved mRNA vaccines. The adverse events were reported in phase I clinical trial (ChiCTR2000039212) of the mRNA vaccine called ARCoV against SARS‐CoV‐2, with an adverse event rate of 100% in the group of 25 µg.^[^ ^22^ ^]^ Therefore, it is urgently needed to develop effective and safe vaccines against the variants of SARS‐CoV‐2.

Highly protective mRNA vaccines also require efficient delivery systems for mRNA. Previous reports have shown that the immune effect of mRNA vaccines is closely associated with the mRNA delivery system.^[^ ^23^ ^]^ Lipid nanoparticle (LNP) systems are currently the leading nonviral delivery systems for enabling the clinical potential of mRNA drugs.^[^ ^24^ ^]^ Notably, the authorized COVID‐19 vaccines, namely mRNA‐1273 and BNT162b2, utilize LNPs to deliver antigen mRNA.^[^ ^1^ ^]^ Nonetheless, there are also many problems in the application of LNP. Excellent LNPs should not only have high translation efficiency but also have an opportune immunoadjuvant property and good safety. Ionizable lipids, the critical components of LNPs, have an important impact on the effectiveness of LNPs, which in turn affect the therapeutic effect of mRNA vaccines.^[^ ^24^, ^25^ ^]^ Ionizable lipids provide positive charges to encapsulate mRNA into LNPs and enable the loaded mRNA to cross the cell membrane. The delivery efficiency of LNP systems can be improved by rational design of the chemical structure of ionizable lipids to increase cellular uptake and endosomal escape.^[^ ^24^, ^26^ ^]^ According to the chemical structure, ionizable lipids can be divided into singly charged lipids and multi‐charged lipids. The ionizable lipids in mRNA‐1273 and BNT162b2 are SM‐102 and ALC‐0315, respectively. Both SM‐102 and ALC‐0315 are singly charged lipids. However, the translation efficiency, immunogenicity, and even safety of mRNA vaccines still need further improvement. A lot of researches have also focused on the multi‐charged lipids,^[^ ^24^ ^]^ such as C12‐200, G0‐C14, cKK‐E12, OF‐2, TT3, and 306O_i10_. When the mass ratios of ionizable lipids to mRNA are the same, multi‐charged lipids usually have higher N/P ratios than singly charged lipids, which is conducive to the encapsulation, uptake, and lysosome escape of mRNA. In this paper, we designed a new set of multi‐charged lipids with four tertiary amino nitrogen atoms (4N4T). The structure of 4N4T ionizable lipids can be divided into a hydrophilic center containing tertiary amines and four hydrophobic tails. The design of 4N4T preserves the differences in the fine structure of different lipids so as to investigate the impact of small structural differences on delivery efficiency and immunogenicity.

**Figure 1:**
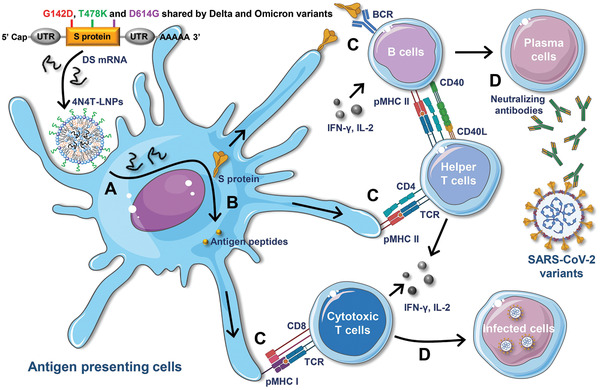
Schematic illustration of the 4N4T‐DS mRNA vaccine against SARS‐CoV‐2 variants. A) DS mRNA is encapsulated in 4N4T ionizable lipid nanoparticles, thus forming 4N4T‐DS mRNA vaccines. LNPs containing DS mRNA are taken in by antigen‐presenting cells after vaccination and DS mRNA is released in the cytoplasm. B) S proteins are translated by ribosomes, processed into antigen peptides, and then integrated on cytomembrane or secreted outside the APCs. C) APCs present antigenic peptides to cytotoxic T cells and helper T cells via the MHC I route and MHC II route, respectively. B cells receive the first signal from S proteins through BCR and the second signal from activated helper T cells via CD40. D) Then, B cells are activated and subsequently differentiate into plasma cells, and S‐specific neutralizing antibodies are produced. Besides, cytotoxic T cells can eliminate the infected cells.

Herein, we designed a DS mRNA containing three key mutations (G142D, T478K, and D614G) in the Omicron variant based on the full‐length S protein of Delta variant and employed it as a model antigen, thus forming second‐generation mRNA vaccines against SARS‐CoV‐2 variants, to test the efficacy of 4N4T lipid‐based delivery system for mRNA (**Figure** **1**). Head‐to‐head comparisons of 4N4T lipids and SM‐102 showed that 4N4T‐DS mRNA vaccines based on MIC1 and MIC2 had higher delivery efficiency and triggered a more robust and durable immune response against variants of SARS‐CoV‐2, including Delta and Omicron. Thus, our study proved that 4N4T‐DS mRNA vaccines have the potential as vaccines against SARS‐CoV‐2 variants. Collectively, these findings provide evidence that 4N4T‐LNPs can be a potent mRNA vaccine platform for infectious diseases and help the development of mRNA drugs as a powerful and versatile tool to combat diseases.

## 2 Results and Discussion

### 2.1 Design of 4N4T Ionizable Lipids and Construction of 4N4T‐LNPs

**Figure 2:**
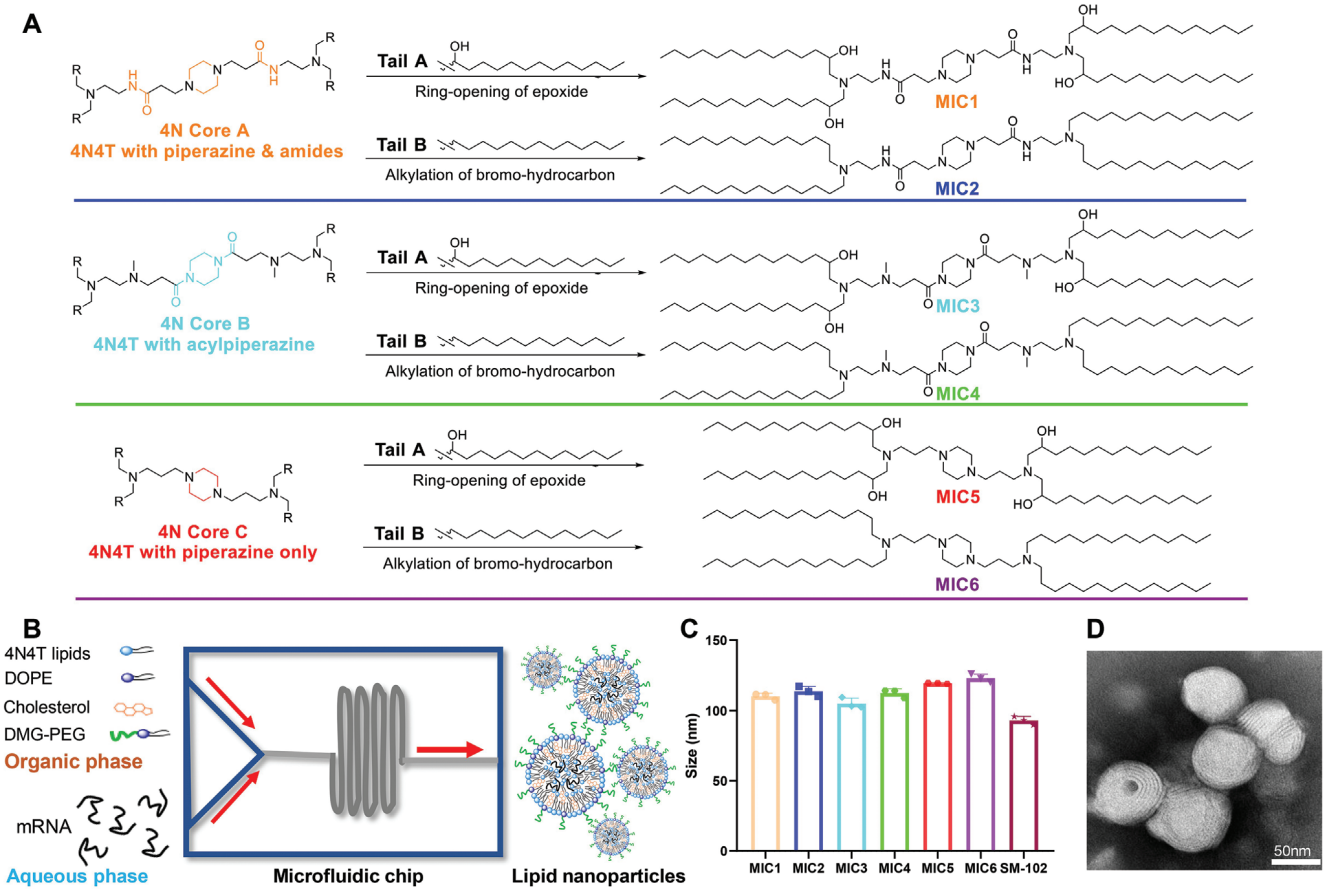
Construction and characterization of 4N4T‐mRNA LNPs. A) Chemical structures of 4N4T ionizable lipids. B) Preparation mechanism of 4N4T‐mRNA LNPs. Briefly, liposoluble alkaline lipids were infused in an acid aqueous solution and the mixture was squeezed through a microfluidic chip. C) Average size of 4N4T‐LNPs measured by DLS (*n* = 3). D) Representative TEM images of MIC1‐LNPs.

Lipid nanoparticles(LNPs) are currently the best choice for nucleic acid drug delivery systems. There have been three approved nucleic acid medicines based on LNPs so far: Patisiran based on DLin‐MC3‐DMA LNPs, BNT162b2 based on ALC‐0315 LNPs, and mRNA‐1273 based on SM‐102 LNPs.^[^ ^1^, ^24^ ^]^ LNPs typically consist of four components including an ionizable lipid, a phospholipid, cholesterol, and a PEGylated lipid, among which the ionizable lipids play the critical role in mRNA loading and differentiate the efficiency of LNPs.^[^ ^24^, ^26^ ^]^ According to the review of reported chemical structures of ionizable lipids used in LNPs for mRNA,^[^ ^27^, ^28^, ^29^, ^30^ ^]^ we designed 4N4T lipids with a positively charged core formed of four tertiary amine nitrogen‐atoms (4N) and four hydrophobic tails (4T) (**Figure** **2**A). To get the compounds, the 4N core was synthesized via a Michael addition reaction and deprotection of Boc groups, and then epoxidated by an epoxide ring‐opening reaction or alkylation reaction of bromo‐hydrocarbon. The synthesis routes and characterization of 4N4T are shown in Figures S1‐S3 and Table S1 (Supporting Information).

4N4T‐LNPs were produced by squeezing the mixture of alcoholic lipid solution and aqueous mRNA solution into the microfluidic chip. The principle of preparation is that amphiphilic lipids form nanostructures with a hydrophilic outer layer and a hydrophobic inner layer in water, thus entrapping mRNA into them (Figure 2B). Detailed formulation and preparation process are shown in the experimental section and the results of formulation screening and optimization are shown in Figure S4 (Supporting Information). The prepared 4N4T‐LNPs were small and uniform, with an average size of ≈100 nm, as measured by dynamic light scattering (DLS) (Figure 2C). The positive potential of LNPs was also moderate (Figure S5, Supporting Information). The formulation properties of 4N4T‐LNPs and SM‐102‐LNPs were comparable. 4N4T‐LNPs showed a solid spherical shape under the transmission electron microscope (TEM) (Figure 2D; Figure S6, Supporting Information). The fingerprint‐like structure was considered a characteristic of LNPs.^[^ ^31^ ^]^

### 2.2 Expression of Reporter Genes Delivered by 4N4T‐LNPs In Vitro and In Vivo

**Figure 3:**
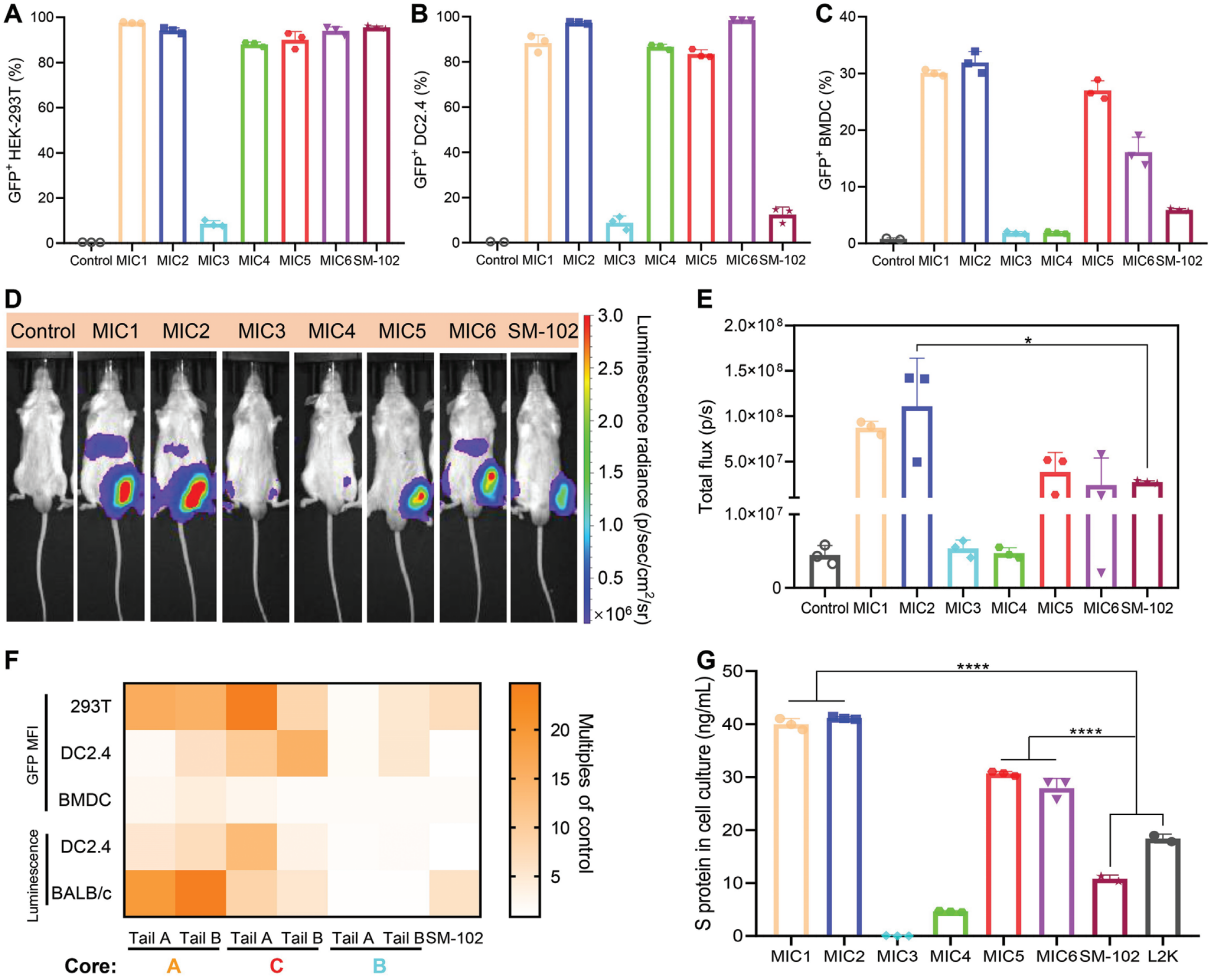
Expression of the reporter gene and DS mRNA delivered by 4N4T‐LNPs. A–C) 293T cells, DC2.4 and BMDCs were transfected with 4N4T‐GFP mRNA and then harvested after 24 h. GFP^+^ cells were analyzed and quantified using flow cytometry. D,E) In vivo expression of FLuc mRNA delivered by 4N4T‐LNPs. Male BALB/c mice were intramuscularly injected with 20 µg of FLuc mRNA encapsulated in 4N4T‐LNPs. Bioluminescence was measured 8 h later in an IVIS imaging system. F) MFI of GFP and value of bioluminescence were all converted into multiples of the negative control. Core A: MIC1, MIC2; Core B: MIC 5, MIC6; Core C: MIC3, MIC4. G) The concentration of S protein in the cell culture was measured using ELISA after 293T cells had been incubated with 4N4T‐DS mRNA vaccines for 24 h. All data are presented as the mean ± SD (*n* = 3). Statistical significance was analyzed by one‐way ANOVA. (ns or unmarked, not significant; *, *p * < 0.05; **, *p*  < 0.01; ***, *p*  < 0.001; ****, *p*  < 0.0001).

To comprehensively evaluate the delivery efficiency of 4N4T‐LNPs, we employed GFP mRNA and firefly luciferase (FLuc) mRNA as reporter genes for in vitro and in vivo tests. HEK293T cells, DC2.4 cells, and BMDCs were chosen for the GFP mRNA transfection test. Cells were cultured to logarithmic growth phase and transfected with GFP mRNA encapsulated in 4N4T‐LNPs or SM‐102‐LNPs at 1 µg mL^–1^ per 10^5^ cells. After 24 h, the cells were collected and analyzed by flow cytometry. For FLuc mRNA transfection, the cells were treated with luciferin and then measured by a microplate reader. Phosphate buffered saline (PBS) was employed as a negative control. Representative fluorescent microscopy images of DC2.4 cells after transfection with 4N4T‐GFP mRNA are shown in Figure S7 (Supporting Information). The GFP+ rates of three cells were counted, as shown in **Figure** **3**A–C. BMDCs were more difficult to transfect than HEK293T cells and DC2.4 cells. Nonetheless, we were surprised to find that several 4N4T‐LNPs showed significantly higher levels of transfection in both antigen‐presenting cells (APCs) than SM‐102‐LNPs. This performance of 4N4T‐LNPs was conducive to their application in vaccines.

Vaccines are usually administrated via intramuscular injection. To test mRNA expression of 4N4T‐LNPs upon intramuscular injection, in vivo expression of FLuc mRNA was conducted in male BALB/c mice. Mice were intramuscularly (i.m.) injected with 20 µg of FLuc mRNA encapsulated in 4N4T‐LNPs or SM‐102‐LNPs. Normal saline (NS) was employed as a negative control. After 8 h, the mice were intraperitoneally (i.p.) injected with luciferin, and bioluminescence signals were collected by an In Vivo Imaging System (IVIS) Spectrum instrument. Representative images in bioluminescence and statistical data are shown in Figure 3D,E, respectively. In addition, we monitored changes in luciferase activity over time (Figure S8, Supporting Information). LNPs of MIC1, MIC2, and MIC5 showed higher expression levels than SM‐102‐LNPs, thus guaranteeing the in vivo expression of 4N4T‐DS mRNA vaccines.

In Figure 3F, we studied the structure‐activity relationship between the expression of reporter genes and the chemical structures of 4N4T‐LNPs. The primary structure of 4N4T is composed of four tertiary amine nitrogen atoms and four hydrophobic tails, but each lipid has its own finer structure. The fine structures of 4N4T lipids can be summarized into three kinds of cores and two kinds of tails, among which Core A consists of “piperazine & amides” (MIC1 and MIC2), Core B consists of “acylated piperazine” (MIC3 and MIC4) and Core C consists of piperazine only (MIC5 and MIC6), as shown in Figure 1A. We next converted the mean fluorescence intensity (MFI) of GFP and the value of bioluminescence into multiples of the negative control. The result showed that the expression level of the reporter gene delivered by 4N4T‐LNPs with Core A was the highest, followed by Core C, and Core B was the weakest. Whether the tail was hydroxyl or not seemed to have no effect on expression. The results indicate that a positively charged core with the structure of “piperazine & amides” was favorable for mRNA expression, but this was not the case when piperazine was amidated as in MIC3 or MIC4. Additionally, 4N4T‐LNPs showed much higher efficiency in expressing the reporter genes than SM‐102‐LNPs, both in vitro and in vivo. The likely reason was that the higher N/P ratio of multi‐charged 4N4T lipids to mRNA assisted the lysosomal escape of LNPs.

### 2.3 Expression of Delta Spike mRNA Encapsulated in 4N4T‐LNPs

Furthermore, the in vitro expression of 4N4T‐DS mRNA was determined by an enzyme‐linked immunosorbent assay (ELISA).^[^ ^32^ ^]^ DS mRNA was designed based on the full‐length Spike protein of Delta variant and synthesized via in vitro transcription.^[^ ^33^ ^]^ 4N4T‐DS mRNA vaccines were prepared and well characterized. HEK293T cells in the logarithmic growth phase were cultured in 10 cm dishes and treated with 20 µg of 4N4T‐DS mRNA per 10^6^ cells. After incubation for 24 h, the supernatant of cell culture was collected and assayed for S protein using an RBD quantification ELISA kit. Standard curve line of the OD value versus the concentration of receptor‐binding domain (RBD) was plotted. Concentrations of S protein in the cell culture were calculated indirectly. (Figure 3G). Expression of 4N4T‐DS mRNA was significantly superior to SM‐102‐LNPs and Lipofectamine 2000 (L2K), which is comparable to the expression of reporter genes.

### 2.4 4N4T‐DS mRNA Vaccines Provoked a Robust and Long‐Lasting Humoral Immune Response

**Figure 4:**
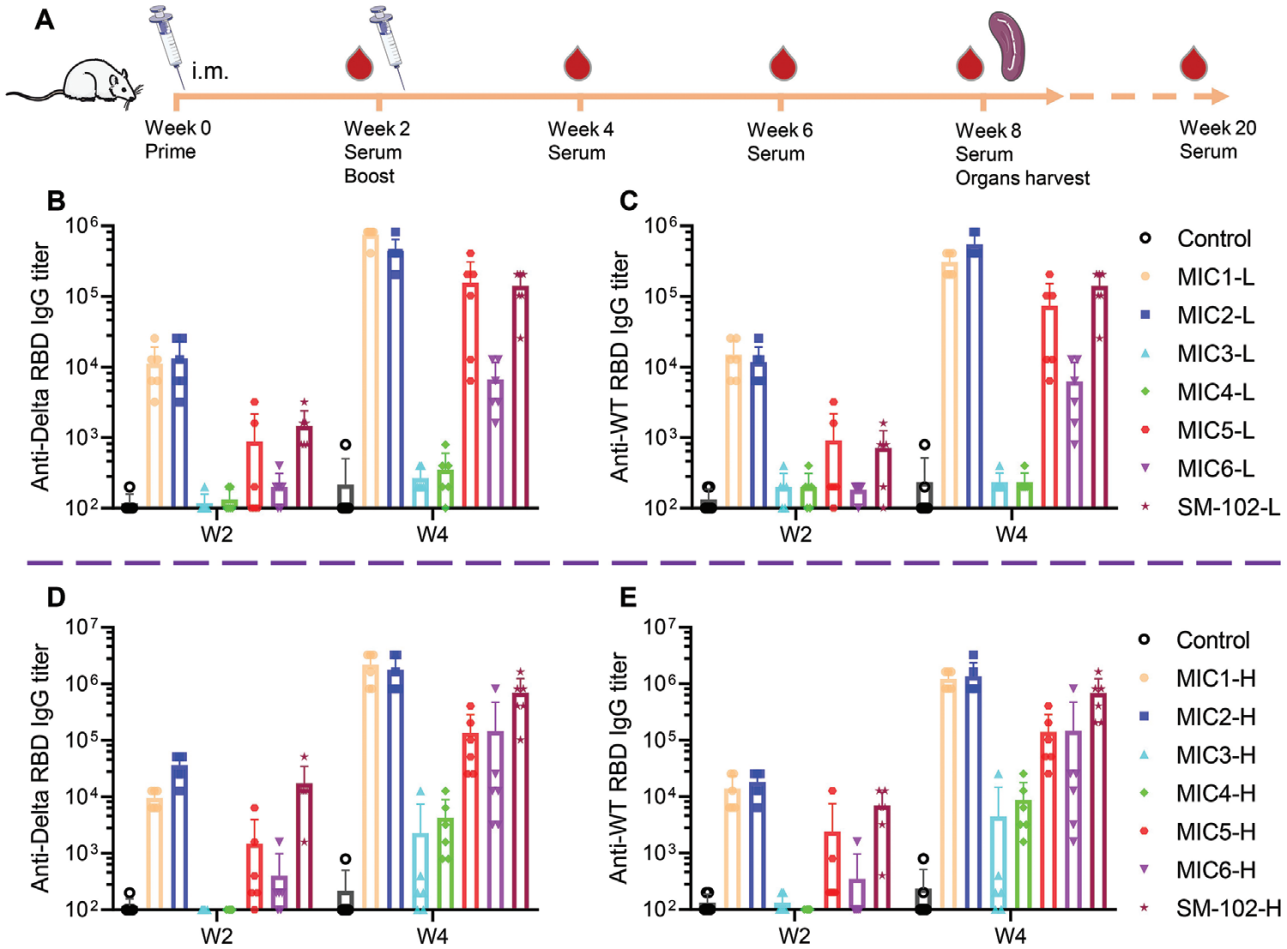
Mouse immunization schedule and humoral immune response evoked by 4N4T‐DS mRNA vaccines. A) Timeline of immunization and sample collection. BALB/c mice were intramuscularly injected twice with 10 or 30 µg of 4N4T‐DS mRNA at week 0 and week 3. Mice in the control group were injected with normal saline. Serum was collected every two weeks and Spike RBD‐specific IgG titers were detected by ELISA. B) Anti‐Delta RBD IgG titer of the low‐dose group. C) Anti‐WT RBD IgG titer of the low‐dose group. D) Anti‐Delta RBD IgG titer of high‐dose group. E) Anti‐WT RBD IgG titer of the high‐dose group. Low‐dose (L) = two doses of 10 µg DS mRNA, high‐dose (H) = two doses of 30 µg DS mRNA.

**Figure 5:**
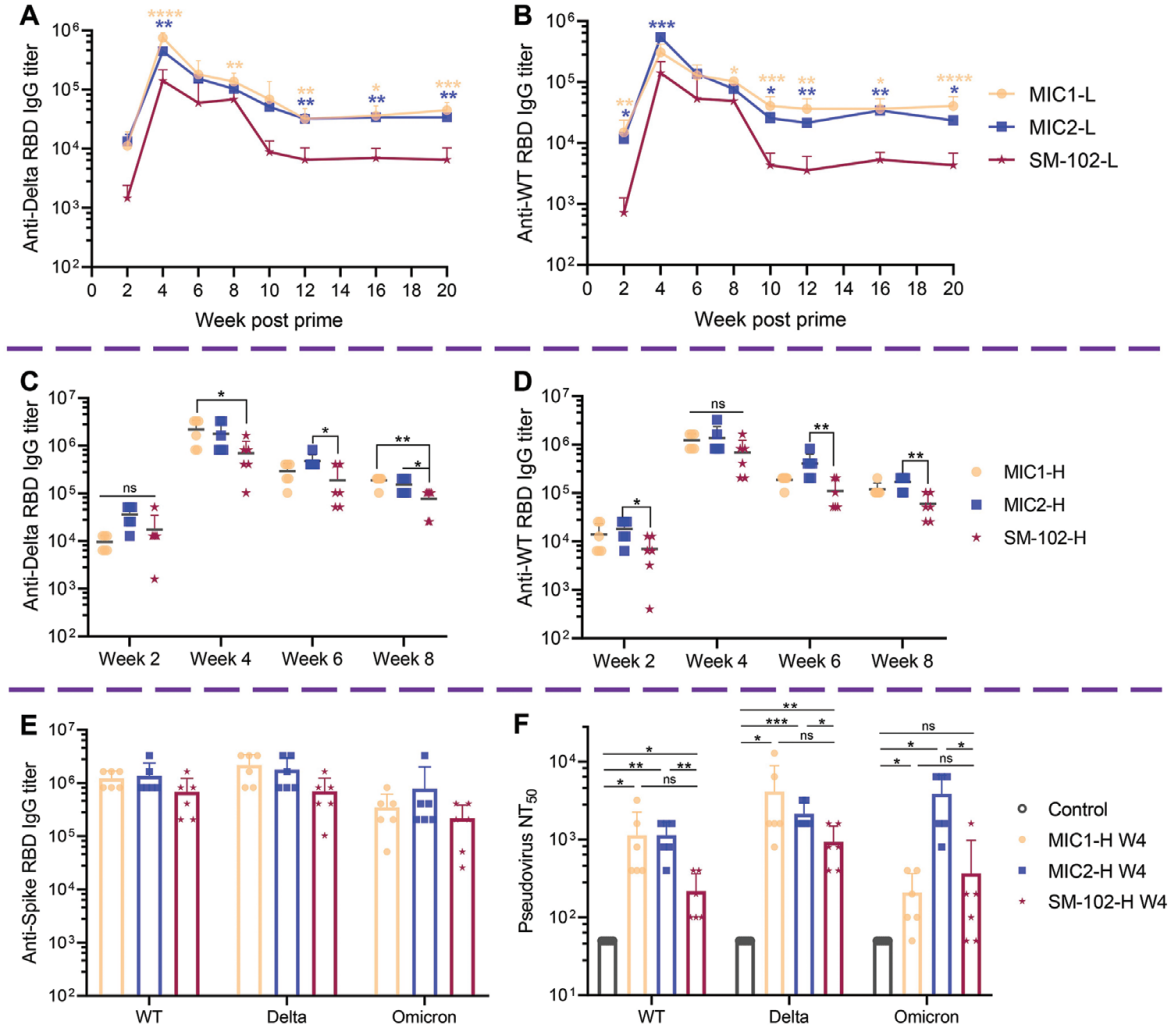
Comparison of humoral immune response between 4N4T‐based vaccines and approved lipid‐based vaccines. A,B) The MIC1‐L and MIC2‐L groups showed significantly higher titers of long‐lasting IgG for both Delta RBD A) and WT RBD B), compared to the SM‐102 group. C,D) There were also differences between the high‐dose groups. E) Anti‐Omicron RBD IgG titers were also tested using serum from week 4 in the high‐dose groups. F) Serum from week 4 in the high‐dose groups was also examined for pseudovirus‐neutralizing antibodies. All data are presented as the mean ± SD (*n* = 6). Statistical significance was analyzed by one‐way ANOVA (A,B,C,D) and Welch's t‐test (F). (ns or unmarked, not significant; *, *p* < 0.05; **, *p* < 0.01; ***, *p* < 0.001; ****, *p* < 0.0001).

Previous work in this paper validated the expression of mRNA encapsulated in 4N4T‐LNPs administrated intramuscularly and the expression of DS mRNA at the cellular level. Next, we initiated experiments on the efficiency of 4N4T‐DS mRNA vaccines against SARS‐CoV‐2 and its variants. Male BALB/c mice were randomly divided into low‐dose (L) and high‐dose (H) groups, and administrated with two doses of 10 and 30 µg of 4N4T‐DS mRNA, respectively. NS was used as the negative control. The timeline of immunization and sample collection is shown in **Figure** **4**A. The serum of immunized mice was collected and analyzed by ELISA.^[^ ^33^ ^]^ The RBD of S protein of both Delta variant and wild‐type (WT) were utilized to detect specific immunoglobulin G (IgG) in serum. Pre‐ and post‐boost anti‐RBD IgG titers in all groups are listed in Figures 4B–E. Specific IgG titers in the high‐dose groups were significantly higher than those in the low‐dose groups, and a second immunization with 4N4T‐DS mRNA resulted in a rapid elevation of specific IgG. Compared to other 4N4T‐DS mRNA vaccines, the MIC1 and MIC2 groups produced much more specific IgG, with the titer of week 4 approaching 10^6^, which was much higher than the reported IgG titer,^[^ ^23^, ^32^, ^34^ ^]^ so we selected them for further research. The high‐dose groups were used for cellular immunology research at week 8, so long‐term humoral immunity maintenance was monitored in the low‐dose groups, as shown in **Figure** **5**A,B. The comparison of IgG titers in the high‐dose groups from week 2 to week 8 is shown in Figure 5C,D. 4N4T‐DS mRNA vaccine‐induced humoral responses peaked two weeks after the boost and then dropped to a platform with a specific IgG titer of ≈5 × 10^4^. As expected, the specific IgG titers of groups MIC1 and MIC2 were consistently significantly higher than that of SM‐102 in both the high‐dose and low‐dose groups.

Besides, the neutralizing antibodies (NAb) were detected using a pseudovirus neutralizing assay.^[^ ^35^, ^36^ ^]^ In brief, pseudoviruses containing the luciferase reporter gene were first neutralized with diluted serum and then incubated with 293T‐ACE2 cells. After 48 h, luciferin was added, and luciferase activity was measured using a microplate reader. The 50% neutralization titer (NT_50_) was defined as the serum dilution at which the luminescence value decreased by 50% compared with the virus control. The NAb titers for wild‐type pseudovirus, Delta pseudovirus, and Omicron pseudovirus are shown in Figure 5F.

The Omicron variant has now replaced the Delta variant as the globally dominant strain. Compared to Delta with 18 amino acid (aa) mutated residues in the S protein, Omicron has 43 aa mutated residues in the S protein, which is now escaping the majority of existing SARS‐CoV‐2 neutralizing antibodies and the Pfizer/BioNTech BNT162b2 neutralization.^[^ ^17^, ^18^, ^37^ ^]^ Herein, we also examined the anti‐Omicron efficiency of DS mRNA vaccines containing three key mutations (G142D, T478K, and D614G) in the Omicron variant. The results are shown in Figure 5E,F. We were excited to find that new mutations in Omicron didn't reduce the specific IgG titer and NAb titer of 4N4T‐DS mRNA vaccines. The likely reason was that DS mRNA covered the mutation sites which assisted Omicron immune escape. Besides, we also found that neutralizing antibodies induced by MIC2‐DS mRNA vaccine had a stronger affinity for Omicron pseudovirus than Delta pseudovirus and wild‐type pseudovirus. In other words, the types of vaccine‐induced neutralizing antibodies appeared to be influenced by the chemical structure of ionizable lipids. Similarly, it was reported that mRNA‐1273 and BNT162b2 induced robust functional humoral immune responses, with differences in epitope recognition and antibody‐mediated functional properties.^[^ ^42^ ^]^ All in all, this work will contribute to the prevention of the spreading Omicron pandemic.

### 2.5 4N4T‐DS mRNA Vaccines Induced Strong Th1‐biased T cell Responses

**Figure 6:**
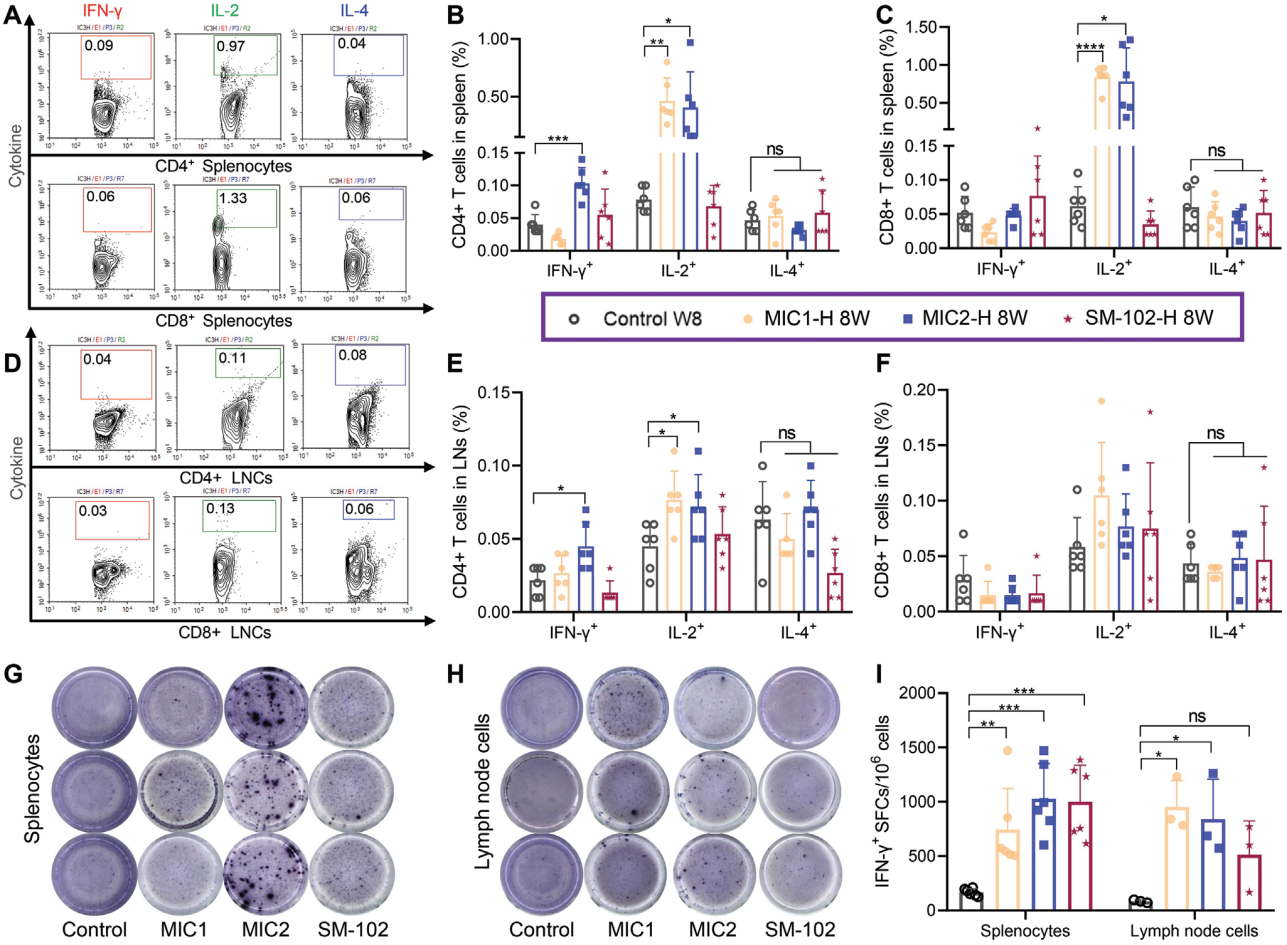
4N4T‐DS mRNA vaccines induced strong Th1‐biased T cell responses. A–F). Major organs of mice in the high‐dose groups were harvested 8 weeks after prime. Isolated splenocytes and lymph node cells (LNCs) were stimulated with a peptide pool of Delta Spike. Then, intracellular staining was performed to quantify Spike‐specific cytokine‐secreting CD4+ and CD8+ T cells. G‐I) The quantification of restimulated IFN‐γ‐secreting T cells in splenocytes and lymph node cells was also verified in the ELISpot test. All data are presented as the mean ± SD. Statistical significance was analyzed by the unpaired t‐test and Welch's t‐test (B, C, E, F), or one‐way ANOVA I). (ns or unmarked, not significant; *, *p* < 0.05; **, *p* < 0.01; ***, *p* < 0.001; ****, *p* < 0.0001).

Helper T cells (Th) are tasked with providing secondary signals during B cell activation and antibody production (Figure 1). Additionally, secretion of immune‐modulatory cytokines such as interferon‐γ (IFN‐γ) results in Th type 1 (Th1)‐biased T cell responses, which aid in countering viral intrusion.^[^ ^38^ ^]^ To examine specific T cell responses induced by 4N4T‐DS mRNA vaccines and the polarization type of T cell responses, intracellular staining (ICS) for IFN‐γ, interleukin (IL)‐2 and IL‐4,^[^ ^34^, ^35^ ^]^ and an enzyme‐linked immunospot (ELISpot) assay for IFN‐γ and IL‐4 were carried out.^[^ ^35^, ^39^, ^40^ ^]^ Mice in the high‐dose groups of MIC1, MIC2, and SM‐102 were euthanized at week 8, and the splenocytes and lymph node cells (LNCs) were collected. The cells were stimulated with a pool of Spike peptides to secrete cytokines. Some stimulated cells were utilized for ICS and flow cytometry to identify cytokine‐producing CD4+ and CD8+ T cells (**Figure** **6**A–F). The other part of stimulated cells was seeded in pre‐coated plates for ELISpot to detect Spike‐specific IFN‐γ‐ or IL‐4‐producing T cells (Figure 6G,I and Figure S9, Supporting Information). According to the results of ICS and ELISpot, spike‐specific CD4+ and CD8+ T cells in both splenocytes and LNCs tended to secrete IFN‐γ, IL‐2, or both, but not IL‐4, indicating that 4N4T‐DS mRNA vaccines induced Th1‐biased T cell responses. The high IgG2a/IgG1 ratio was also consistent with this conclusion (Figure S10, Supporting Information).^[^ ^34^ ^]^

### 2.6 Preliminary In Vivo Safety Evaluation of 4N4T‐DS mRNA Vaccines

Although it is urgent to develop vaccines against SARS‐CoV‐2 variants, safety is still a prerequisite in the development of vaccines. Delivery systems, especially ionizable lipids, are often considered one of the safety risks of mRNA vaccines. In this article, we preliminarily evaluated the in vivo safety of 4N4T‐DS mRNA vaccines. Male BALB/c mice were i.m. administrated with 30 µg of 4N4T‐DS mRNA and serum was used to examine the blood biochemistry indexes 24 h after the administration (Figure S11, Supporting Information). The results showed that there was no significant difference in liver and kidney function between the treated groups and the NS control. At week 8, all the mice in the high‐dose group were sacrificed and the major organs were excised and examined via H&E staining (Figure S12, Supporting Information). No difference among the H&E‐stained histopathological tissues was found. These results demonstrated that 4N4T‐LNPs could be used for safe mRNA vaccine delivery.

## 3 Conclusion

With the approval of two mRNA vaccines, mRNA is becoming more and more widely used, which puts forward higher requirements for mRNA delivery systems.^[^ ^41^ ^]^ Facing the global pandemic of SARS‐CoV‐2 variants, the efficacy of approved mRNA has been challenged and the safety is also being questioned, so it is urgent to develop second‐generation vaccines against SARS‐CoV‐2 variants with high efficiency of immune protection and good safety. To solve these problems, we constructed novel 4N4T‐LNPs for mRNA delivery and applicated them in mRNA vaccines against SARS‐CoV‐2 variants in this paper. Firstly, a set of novel ionizable lipids with multiple charges named 4N4T were designed and synthesized and then used to form 4N4T‐LNPs with good pharmaceutical properties. Next, the delivery efficiency of the 4N4T‐LNPs was examined in vitro and in vivo. MIC1 and MIC2 with the core of “piperazine & amides”, which expressed mRNA more efficiently than SM‐102, were selected. Furthermore, we determined the immunogenicity and efficacy of 4N4T‐DS mRNA vaccines, of which the mRNA was designed on the basis of the full‐length S protein of Delta variant and contained three key mutation sites shared with Omicron variant. Leading 4N4T‐DS mRNA vaccines successfully induced a robust and long‐lasting humoral response in mice, in which the RBD‐specific IgG titer and the neutralizing antibody titer were both higher than those of the SM‐102‐based vaccine. Moreover, antibodies stimulated by 4N4T‐DS mRNA vaccines were effective against all three pseudoviruses of wild‐type, Delta, and Omicron. Additionally, 4N4T‐DS mRNA vaccines also induced a strong Th1‐skewed T cell response with substantial secretion of type‐1 cytokines, namely, IFN‐γ and IL‐2. Specific T cells can assist the humoral response and also confer long‐lasting immune memory. In addition, we were surprised to find that the types of vaccine‐induced neutralizing antibodies could be influenced by the chemical structure of ionizable lipids. We speculate that this result may be related to the adjuvant properties of ionizable lipids, which need further study.

Overall, the 4N4T‐LNPs were successfully applied to DS mRNA vaccine and the vaccines worked well against SARS‐CoV‐2 and its variants, including Delta and Omicron. The result indicated that the 4N4T‐based delivery system would contribute to the application of mRNA vaccines against infectious diseases.

## 4 Experimental Section

### Synthesis of 4N4T lipids

The synthesis routes were shown in Figures S1–S3 (Supporting Information). MIC1 and MIC2 were taken as samples to introduce the synthesis of 4N4T lipids. All reagents used were analytically pure. To a solution of *N*‐Boc‐ethylenediamine (10 mmol, 1 eq) and triethylamine (20 mmol, 2 eq) in anhydrous dichloromethane (DCM) was dropwise added acrylyl chloride (12 mmol, 1.2 eq) on ice. The mixture was stirred in an ice‐water bath overnight and the crude product was purified by silica gel column chromatography to yield Compound 1. A mixture of Compound 1 (8 mmol, 2 eq) and piperazine (4 mmol, 1 eq) in ethanol was stirred at 60 °C overnight and then evaporated under vacuum. The crude product was purified by silica gel column chromatography to yield Compound 2. Compound 2 (4 mmol) was added to 4 mL of trifluoroacetic acid in 8 mL of DCM and stirred at room temperature for 2 h. The product was evaporated under a vacuum and divided into two equal parts. To the solution of deprotected Compound 2 (2 mmol, 1 eq) in isopropanol was added sufficient K_2_CO_3_ followed by 2‐dodecyloxirane (12 mmol, 6 eq) and stirred at 90 °C for 24 h. The product was filtered and evaporated under vacuum, and then purified by column chromatography to obtain MIC1. To the solution of deprotected Compound 2 (2 mmol, 1 eq) in isopropanol was added sufficient K_2_CO_3_ followed by 1‐bromotetradecane (12 mmol, 6 eq) and stirred at 80 °C for 48 h. The product was filtered and evaporated under vacuum, and then purified by column chromatography to obtain MIC2. Besides, the synthesis of SM‐102 was entrusted to HITGEN (Figure S13, Supporting Information).

### Production of mRNA

The SARS‐CoV‐2 B.1.617.2 lineage (GenBank OK091006.1) was employed as the reference amino acid sequence alignment of Spike. The mRNA was transcribed in vitro (IVT) using T7 RNA polymerase‐mediated transcription from a linearized DNA template containing the optimal codons of S protein.

### Preparation and Characterization of 4N4T‐LNPs

4N4T lipid, DOPE (AVT), cholesterol, and DMG‐PEG2000 were co‐dissolved in alcohol at a mol ratio of 35/16/46.5/2.5. mRNA was diluted in 10 × 10^−3^ m sodium citrate buffer (pH 6.0). The alcohol and aqueous phases were mixed at a 1:3 ratio in a microfluidic chip device using syringe pumps with an mRNA to 4N4T lipid ratio of 15:1 (w/w). The resultant LNPs were filtered with 10 × 10^−3^ m sodium citrate buffer (pH 6.0) against an ultrafiltration membrane at a certain gas pressure and the final concentration of mRNA was 0.1 mg mL^–1^. For SM‐102‐LNPs, the mol ratio of lipids was 50:10:38.5:1.5 and the mRNA to lipid weight ratio was 0.05. The prepared LNPs were diluted tenfold and detected for particle size and potential using a Mastersizer (Malvern).

### mRNA Transfection

GFP or FLuc mRNA‐encapsulated 4N4T‐LNPs were prepared for mRNA transfection tests in vitro. HEK293T and DC2.4 cells were cultured to logarithmic growth phase in complete DMEM (Cytiva) and RPMI 1640 (Cytiva) medium supplemented with 10% (V/V) FBS (Gibco) and 1% (V/V) antibiotics of P/S (HyClone), respectively. BMDCs were induced from bone marrow cells using GM‐CSF (Abcam) and cultured in a complete RPMI 1640 medium. Cells were seeded into 24‐well plates at 100 000 (BMDCs: 500 000) per well and transfected with 1 µg of GFP mRNA encapsulated in 4N4T‐LNPs. The plates were imaged by a fluorescence microscope (OLYMPUS) and the cells were analyzed by a flow cytometer (ACEA NovoCyte). For the transfection of FLuc mRNA, 150 000 DC2.4 cells were incubated with 1 µg of FLuc mRNA encapsulated in 4N4T‐LNPs in 24‐well plates for 24 h and detected in a microplate reader (TECAN) after the addition of 150 µg of luciferin.

### Expression of Firefly Luciferase mRNA In Vivo

FLuc mRNA‐encapsulated 4N4T‐LNPs were prepared and examined. Male BALB/c mice were intramuscularly injected with 20 µg of 4N4T‐FLuc mRNA. After 8 h, the mice were intraperitoneally injected with 3 mg of luciferin (YEASEN) dissolved in 200 µL of PBS. Then, the mice were anesthetized using isoflurane (RWD) and imaged in an In Vivo Imaging System (IVIS) Spectrum instrument (PerkinElmer) 15 min after injection of substrate. To plot the curve of luciferase activity over time, the mice were injected with the substrate and imaged at different time points. The animal experiments in this study have been approved by the Institutional Animal Care and Use Committee (IACUC) of West China Hospital, Sichuan University.

### Quantification of S Protein Expression

S protein expression was detected using a SARS‐CoV‐2 RBD detection ELISA kit (Vazyme) and calculated indirectly via RBD expression. This assay is based on a double‐antibody sandwich to determine the target protein. HEK293T cells were seeded in 10 cm dishes and cultured to the logarithmic growth phase. The cells were then incubated with 20 µg of 4N4T‐DS mRNA per 106 cells for 24 h. Afterward, the supernatant of the cell culture was collected. Samples and standards were added to the antibody pre‐coated wells and incubated at 37 °C for 1 h. After washing with washing buffer, the wells were incubated with another antibody conjugated of HRP at 37 °C for 1 h. Then, the plate was incubated with 3,3′,5,5′‐tetramethylbenzidine (TMB) at 37 °C for 1 h, and the absorbance at 450 nm was measured in a microplate reader (TECAN). A standard curve of OD value versus concentration of RBD was fit with a linear equation and the S protein concentrations in cell culture were calculated indirectly via RBD expression.

### Enzyme‐Linked Immunosorbent Assay (ELISA)

ELISA plates were coated with 0.1 µg of SARS‐CoV‐2 wild‐type RBD, Delta RBD, or Delta RBD recombinant protein (Vazyme) in coating buffer (Sangon Biotech) at 4 °C overnight. The coated plates were washed with washing buffer and blocked with 2% BSA (BioFroxx) in washing buffer at 25 °C for 4 h. Serum samples serially diluted with washing buffer containing 0.2% BSA were added and incubated at 4 °C overnight. Then, the plates were washed and incubated with anti‐mouse IgG‐HRP (Cell Signaling), IgG1‐HRP (Abcam), or IgG2a‐HRP (Abcam) antibodies diluted in a washing buffer containing 0.2% BSA (1:50 000) at 25 °C for 2 h. After washing, TMB (Solarbio) was used for development. Reactions were stopped with 2 m sulfuric acid and the absorbance was measured at 450 nm using a microplate reader (TECAN). The endpoint titer was defined as the dilution fold of serum that exceed the values of the control.

### Pseudovirus Neutralization Assay

SARS‐CoV‐2 Spike pseudotyped virus, SARS‐COV‐2 Spike(B.1.617.2) pseudotyped virus, and SARS‐COV‐2 Spike(B.1.1.529) pseudotyped virus were purchased from Genomeditech. 293T‐ACE2 cells were cultured in complete DMEM at 37 °C in a 5% CO_2_ environment. Serum samples serially diluted with the complete medium were added to white 96‐well plates. A total of 0.1 µL of pseudovirus diluted with 50 µL of complete medium was added to each well. The mixture of serum and pseudovirus was incubated at 37 °C for 1 h. Then, 15 000 293T‐ACE2 cells were added to each well and incubated at 37 °C for 48 h. Afterward, 100 µL of luciferase substrate (Vazyme) was added to each well and shaken for 2 min. Luminescence was measured using a microplate reader (TECAN). The neutralization titer (NT) was defined as the dilution fold of serum necessary for 50% inhibition of luciferase activity compared to the virus control.

### Intracellular Cytokine Staining (ICS) Assay

An ICS assay was conducted to detect Spike‐specific CD4+ and CD8+ immune responses in vaccinated mice. Splenocytes and lymph node cells were separated in complete RPMI 1640 medium (Cytiva) containing 10% FBS and 1% P/S and seeded in 24‐well plates. Then, the cells were incubated with the peptide pool of Delta S protein (DG peptides) (2 µg mL^–1^ of individual peptide) overnight and monensin (YEASEN) was added at a concentration of 2 × 10^−6^ m 2 h after the start of stimulation. After simulation, the cells were collected and stained with anti‐CD4 FITC (Biolegend) and anti‐CD8a APC (Biolegend). Afterward, the cells were fixed and permeabilized in Fixation Buffer (Biolegend) and Intracellular Staining Perm Wash Buffer (Biolegend) successively and then stained with anti‐IFN‐γ PE/Cy7, anti‐IL‐2 PE, and anti‐IL‐4 PerCP/Cy5.5. Flow cytometric analysis and cell sorting were performed on a flow cytometer (ACEA NovoCyte).

### Enzyme‐Linked Immunospot (ELISpot) Assay

ELISpot was a more intuitive way to characterize Spike‐specific T cell responses. Mouse IFN‐γ ELISpot^PLUS^ kit and Mouse IL‐ ELISpot^PLUS^ kit were purchased from MabTech. According to the protocol, the pre‐coated plates with antibodies specific for IFN‐γ or IL‐4 were washed with PBS and incubated with RPMI 1640 medium containing 10% FBS at room temperature for at least 30 min. A total of 500 000 splenocytes or lymph node cells stimulated with the peptide pool were added to each well. The cells were cultured at 37 °C for 48 h. After washing with PBS, the plates were successively incubated with biotinylated anti‐IFN‐γ, or IL‐4 antibody diluted in PBS‐0.5% FBS and streptavidin‐ALP diluted in PBS‐0.5% FBS at room temperature. Spots were exposed following the addition of substrate solution and rinsed in tap water. The plates were naturally dried in the shade and read by an ELISpot reader (AID).

### Statistical Analysis

All values were expressed as mean ± S.D. unless otherwise noted. Statistical analysis was performed using GraphPad Prism 8.0.1. Statistical significance was analyzed by the unpaired t‐test, Welch's t‐test, or one‐way ANOVA. (*p*‐Value: ns or unmarked, not significant; *, *p* < 0.05; **, *p* < 0.01; ***, *p* < 0.001; ****, *p* < 0.0001.)

## Conflict of Interest

The authors declare no conflict of interest.

## Supporting information

Supporting information

## Data Availability

The data that support the findings of this study are available from the corresponding author upon reasonable request.
