# Supplementary material for: mRNA Vaccines Against SARS‐CoV‐2 Variants Delivered by Lipid Nanoparticles Based on Novel Ionizable Lipids
Source: Adv Funct Mater. 2022 Jul 19;32(39):2204692. doi: 10.1002/adfm.202204692 (PMC9349794; doi:10.1002/adfm.202204692)
Supplement: Supplementary file 1 — Supporting information [file ADFM-32-0-s001.pdf]

ADVANCED FUNCTIONAL MATERIALS

Supporting Information

for *Adv. Funct. Mater.,* DOI: 10.1002/adfm.202204692

mRNA Vaccines Against SARS-CoV-2 Variants Delivered by Lipid Nanoparticles Based on Novel Ionizable Lipids

*Kepan Chen, Na Fan, Hai Huang, Xin Jiang, Shugang Qin, Wen Xiao, Qian Zheng, Yupei Zhang, Xing Duan, Zeyi Qin, Yongmei Liu, Jun Zeng, Yuquan Wei, and Xiangrong Song**

mRNA Vaccines against SARS-CoV-2 Variants Delivered by Lipid Nanoparticles Based on Novel Ionizable Lipids

Kepan Chen [#, 1], Na Fan [#, 1], Hai Huang [#, 1], Xin Jiang [#, 1], Shugang Qin [1], Wen Xiao [1], Qian Zheng [1], Yupei Zhang [1], Xing Duan [1], Zeyi Qin [2], Yongmei Liu [1], Jun Zeng [1], Yuquan Wei [1], Xiangrong Song*[, 1]

[1] Department of Critical Care Medicine, Frontiers Science Center for Disease-related Molecular Network, State Key Laboratory of Biotherapy, West China Hospital, Sichuan University, Chengdu 610041, China

[2] Department of Biology, Brandeis University, Boston 02453, USA

E-mail: songxr@scu.edu.cn

Supporting figures

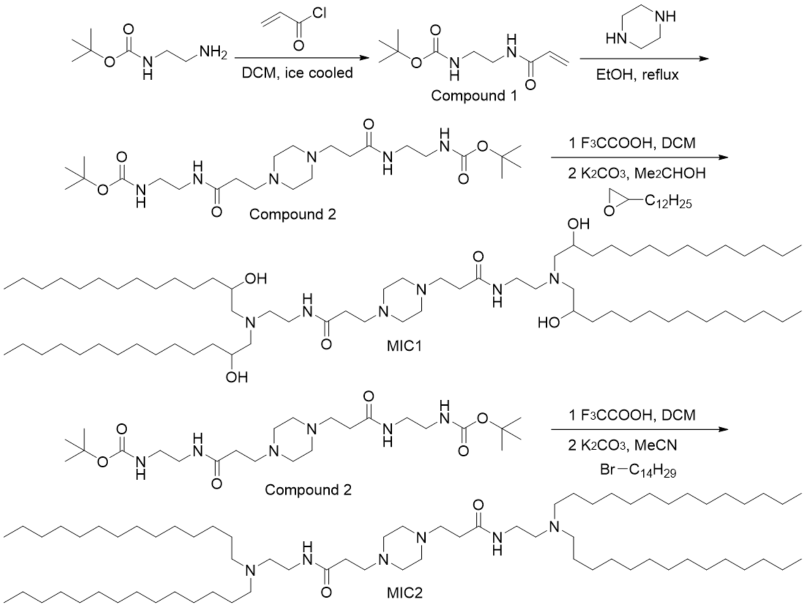

Figure S1. Synthesis routes of MIC1 and MIC2.

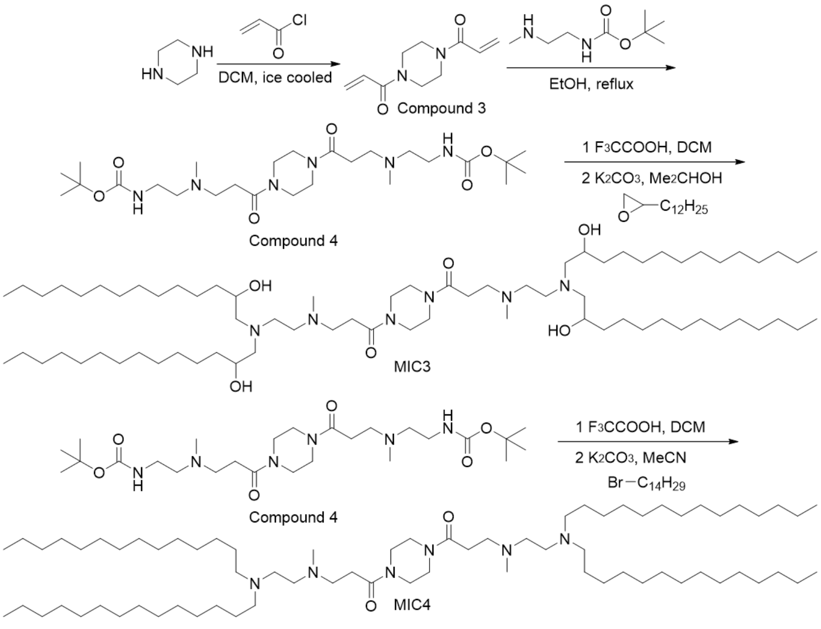

Figure S2. Synthesis routes of MIC3 and MIC4.

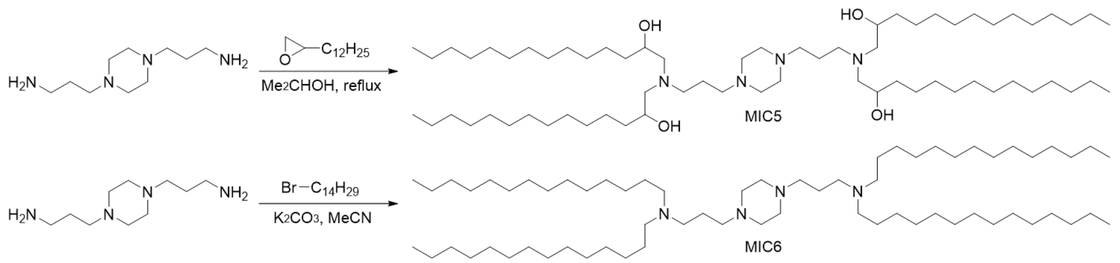

Figure S3. Synthesis routes of MIC5 and MIC6.

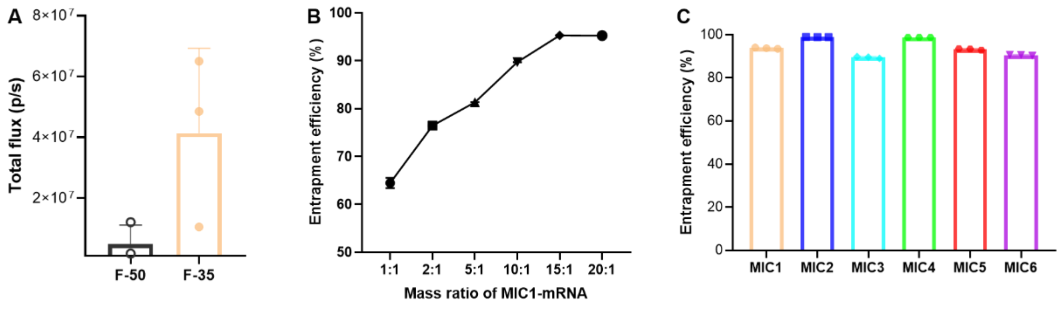

Figure S4. Formulation screening and optimization of 4N4T-LNPs.

A) MIC1-LNPs@FLuc mRNA were prepared using the two formulas reported in literatures, namely 50:10:38.5:1.5[1] (F-50) for MC3 and 35:16:46.5:2.5[2] (F-35) for C12-200 and OF-2[3]. The relative luciferase expression via intramuscular injection (10 μg of mRNA per mouse) was shown in the figure above. Since the fluorescence intensity of the F-35 formula was much higher than that of the F-50 formula, we selected F-35 formula for further research. B) Entrapment efficiency at different mass ratios of mRNA to MIC1 was investigated and 15:1 was the optimized mass ratio. C) Entrapment efficiency of different 4N4T-LNPs prepared using F-35 and mass ratio of 15:1 was examined. The results showed that different LNPs had good entrapment efficiency of mRNA. In summary, we believe that this formula can be universal for the construction of 4N4T-LNPs.

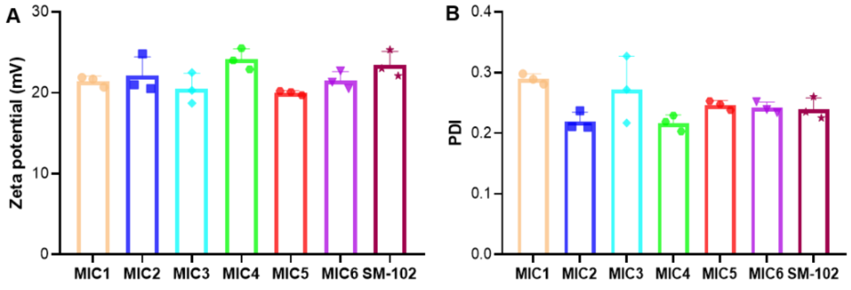

Figure S5. Average ζ potential (A) and PDI (B) of 4N4T-LNPs.

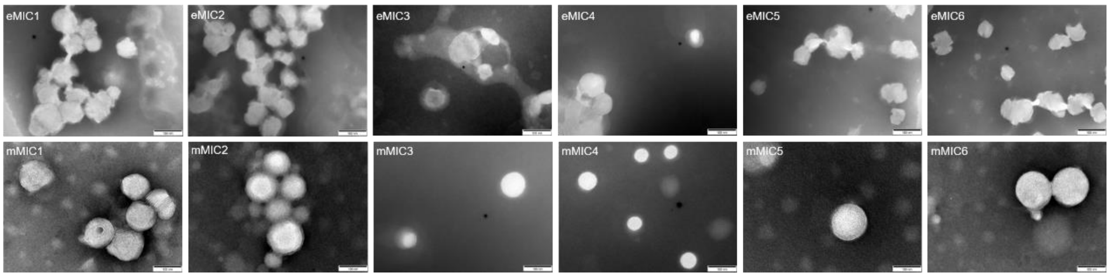

Figure S6. Representative TEM images of 4N4T-LNPs with no mRNA loaded (eMICx) and 4N4T-LNPs loaded with mRNA (mMICx). LNPs of eMICx appeared looser, while LNPs of mMICx were more compact. This should be caused by the compression of ionizable lipids by long-chain mRNA molecules. Scale bar = 100 nm.

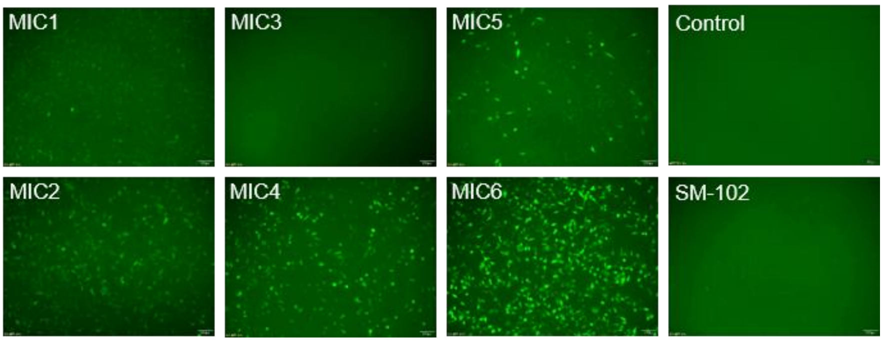

Figure S7. Representative fluorescence microscopic images of DC2.4 cells after 24 h of incubation with 4N4T-GFP mRNA. Scale bar = 100 μm.

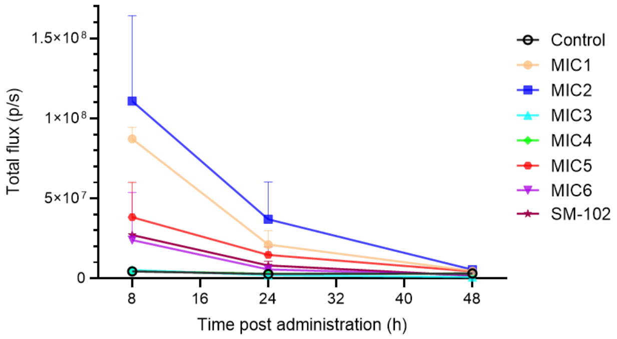

Figure S8. Luciferase activity over time after administration of 10 μg of FLuc mRNA.

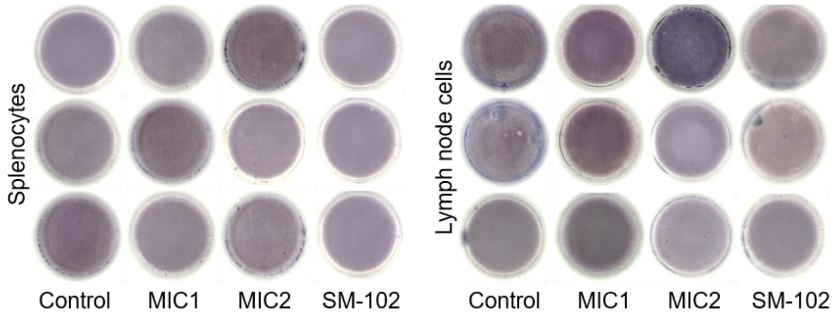

Figure S9. The quantification of IL-4-secreting T cells in splenocytes and lymph node cells.

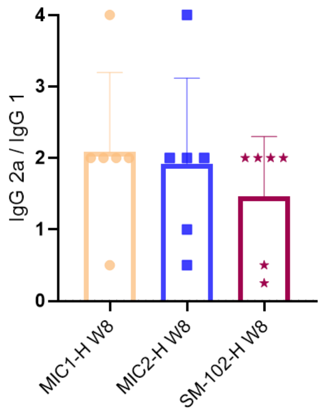

Figure S10. IgG2a/IgG1 ratio of serum in high-dose groups at week 8.

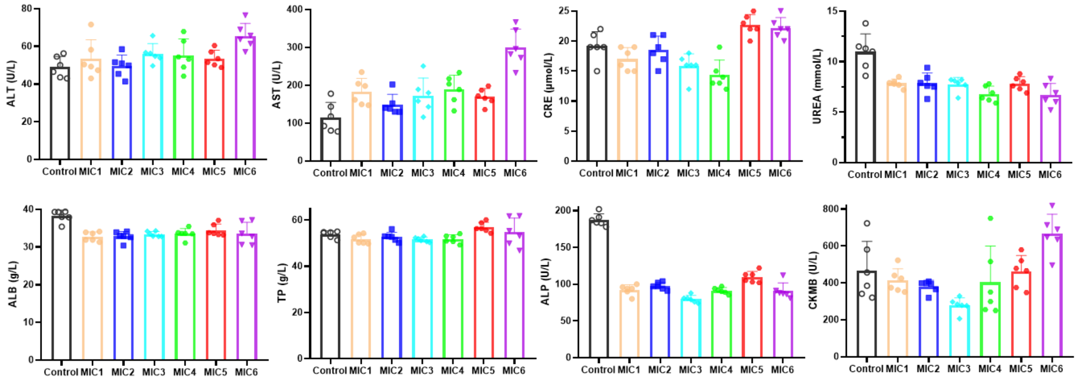

Figure S11. Blood biochemistry of mice 24 h after intramuscular administration with 30 μg 4N4T-DS mRNA.

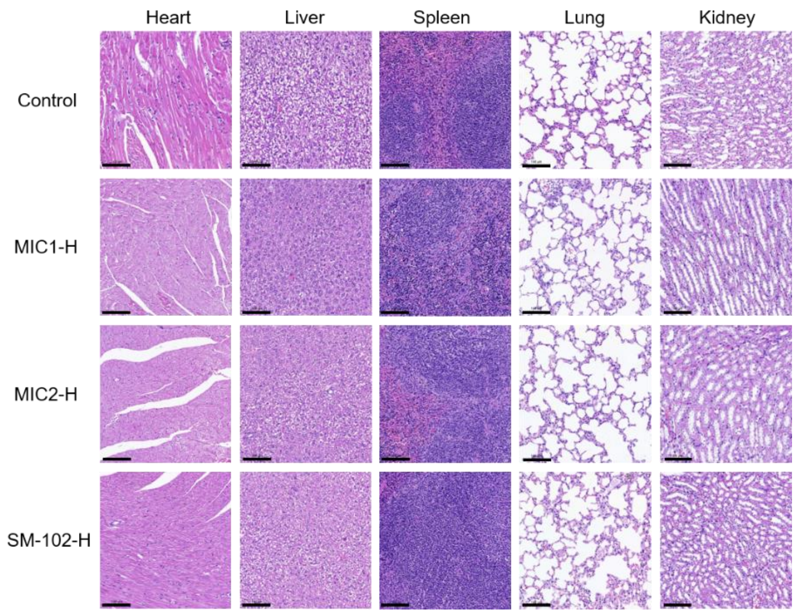

Figure S12. Representative histopathology (H&E) of major organs harvested 8 weeks after vaccination. Scale bar = 100 μm.

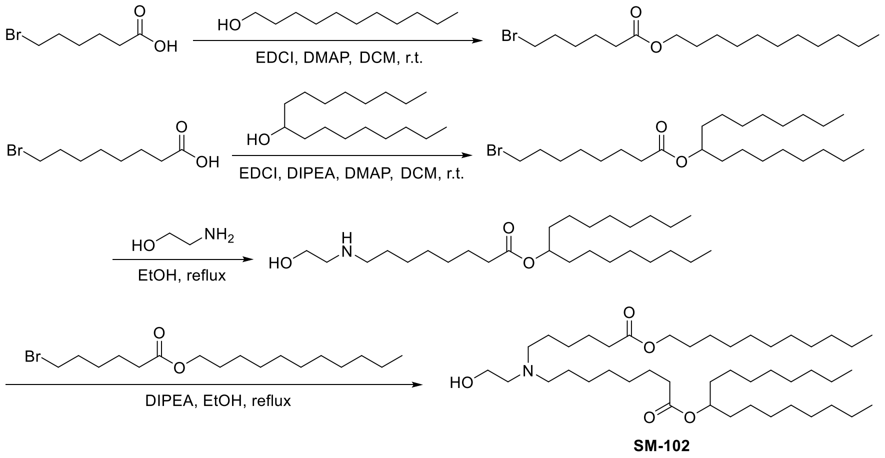

Figure S13. Chemical structure and synthetic route of SM-102.

Table S1. Characterization of 4N4T lipids

Abbreviation	^{1}H NMR (400 MHz, CDCl3, δ)
Compound 1	δ (ppm) = 6.45 (s, 1H), 6.26 (d, J=17.0, 1H), 6.10 (dd, J=17.0, 10.3, 1H), 5.64 (d, J=10.3, 1H), 4.96 (s, 1H), 3.44 (dd, J=11.1, 5.4, 2H), 3.38–3.21 (m, 2H), 1.44 (s, 9H).
Compound 2	δ (ppm) = 8.13 (s, 2H), 4.96 (s, 2H), 3.35 (dd, J=11.6, 5.7, 4H), 3.19 (dd, J=42.8, 6.3, 4H), 2.83–2.45 (m, 12H), 2.39 (t, J=6.1, 4H), 1.40 (s, 18H).
MIC1	δ (ppm) = 8.21–8.01 (m, 2H), 3.58 (s, 4H), 3.41–3.22 (m, 4H), 2.76–2.30 (m, 28H), 1.49–1.37 (m, 8H), 1.26 (s, 88H), 0.88 (t, J=6.8, 12H).
MIC2	δ (ppm) = 7.69 (t, J=4.7, 2H), 3.29 (dd, J=11.2, 5.7, 4H), 2.62 (t, J=6.4, 4H), 2.57–2.28 (m, 24H), 1.40 (m, 8H), 1.35–1.17 (s, 88H), 0.88 (t, J=6.8, 12H).
Compound 3	δ (ppm) = 6.64–6.51 (m, J=16.7, 5.6, 4.6, 2H), 6.33 (dd, J=16.8, 1.7, 2H), 5.76 (dd, J=10.5, 1.7, 2H), 3.70 (d, J=34.4, 8H).
Compound 4	δ (ppm) = 5.20 (s, 2H), 3.58 (dd, J=53.6, 16.8, 8H), 3.22 (d, J=5.0, 4H), 2.79–2.68 (m, 4H), 2.50 (dd, J=12.4, 6.1, 8H), 2.25 (s, 6H), 1.44 (s, 18H).
MIC3	δ (ppm) = 5.19 (s, 2H), 3.75–3.42 (m, 8H), 3.23 (d, J=5.0, 4H), 2.88–2.16 (m, 30H), 1.47–1.16 (m, 92H), 0.89 (t, J=6.8, 12H).
MIC4	δ (ppm) = 3.57 (dd, J=48.8, 18.3, 8H), 2.86–2.21 (m, 30H), 1.55–1.18 (m, 96H), 0.89 (t, J=6.8, 12H).
MIC5	δ (ppm) = 4.61–3.77 (m, 4H), 3.73–3.49 (m, 4H), 2.91–2.14 (m, 24H), 1.68–1.53 (m, 4H), 1.50–1.38 (m, 8H), 1.34–1.23 (s, 88H), 0.88 (t, J=6.8, 12H).
MIC6	δ (ppm) = 2.79–2.30 (m, 24H), 1.76–1.65 (m, 4H), 1.54–1.43 (m, 8H), 1.34–1.18 (s, 88H), 0.88 (t, J=6.1, 12H).

References:

[1] Miao, L.; Lin, J.; Huang, Y.; Li, L.; Delcassian, D.; Ge, Y.; Shi, Y.; Anderson, D. G., Synergistic lipid compositions for albumin receptor mediated delivery of mRNA to the liver. Nat Commun 2020, 11 (1), 2424.

[2] Kauffman, K. J.; Dorkin, J. R.; Yang, J. H.; Heartlein, M. W.; DeRosa, F.; Mir, F. F.; Fenton, O. S.; Anderson, D. G., Optimization of Lipid Nanoparticle Formulations for mRNA Delivery in Vivo with Fractional Factorial and Definitive Screening Designs. Nano Lett 2015, 15 (11), 7300-6.

[3] Fenton, O. S.; Kauffman, K. J.; McClellan, R. L.; Appel, E. A.; Dorkin, J. R.; Tibbitt, M. W.; Heartlein, M. W.; DeRosa, F.; Langer, R.; Anderson, D. G., Bioinspired Alkenyl Amino Alcohol

Ionizable Lipid Materials for Highly Potent In Vivo mRNA Delivery. Adv Mater 2016, 28 (15), 2939-43.
